# Solvent Extraction for Separation of Indonesian Oil Sands

**DOI:** 10.3390/ijerph20054527

**Published:** 2023-03-03

**Authors:** Wenlong Cui, Qingqing Zhu, Chenze Zhao, Weiyou Zhou, Cheli Wang

**Affiliations:** 1 Advanced Catalysis and Green Manufacturing Collaborative Innovation Center, School of Petrochemical Engineering, Changzhou University, Changzhou 213164, China; 2 Zhejiang Chemical Products Quality Inspection Company Ltd., Hangzhou 310023, China

**Keywords:** Indonesian oil sands, solvent extraction, extraction conditions, bitumen analysis

## Abstract

Based on the examination of the basic properties, the solvent extraction process (SEP) was applied with high efficiency in the extraction of bitumen from Indonesian oil sands. To separate the oil sands, different organic solvents were first screened, and the extraction effects were analyzed to select a suitable solvent. Then, the effects of operating conditions on the extraction rate of bitumen were investigated. Finally, the compositions and structures of the bitumen obtained under suitable conditions were analyzed. The results showed that the Indonesian oil sands were oil-wet oil sands with a bitumen content of 24.93%, containing a large number of asphaltenes and resins with high polarity and complex structures. The separation performance was affected by different organic solvents and operating conditions. It was shown that the closer the structure and polarity of the selected solvent is to the solute, the better the extraction effect. The extraction rate of bitumen reached 18.55% when toluene was used as the extraction solvent under the operating conditions of V (solvent):m (oil sands) 3:1, temperature 40 °C, stirring velocity 300 r/min, time 30 min. The method could also be applied to the separation of other oil-wet oil sands. The compositions and structures of bitumen can guide the separation and comprehensive use of industrial oil sands.

## 1. Introduction

With diminishing petroleum reserves and increasing demand, the huge energy gap will be largely compensated by oil sands, shale oil, and other resources in the future. Oil sands are unconventional resources consisting of sands, bitumen, water, and clay, which are abundant worldwide and far exceed proven petroleum reserves [1]. With the decreasing use of conventional resources, and as an important alternative energy source to conventional petroleum, oil sands have drawn the close attention of researchers to the efficient separation and utilization of oil sands. On the other hand, the deposition and accumulation of polycyclic aromatic compounds in the environment rise with the increase of oil sands’ industrial activities, and the concentration levels of SO_2_ and NO_2_ in the air also increase significantly with the extraction of oil sands. The improvement of oil sands’ treatment methods is necessary to effectively limit pollution within the specified thresholds [2]. In addition, crude oil spills contaminate the soil and sandy soil while also seriously affecting the environment and human health. In oil-contaminated sand, the oil easily adheres to the sand, which makes it difficult to clean up. Therefore, the development and application of an effective technology to separate oil from oil sands can solve this major environmental problem [3,4].

Oil sands can be divided into water-wet, oil-wet, and neutral types based on the different wettability and adsorption forms of the sand surface. The selection of oil sand separation methods is limited by the type of oil sand wettability [5]. For the oil sands mixture, the commonly used methods include the pyrolysis process, solvent extraction process (SEP), water-based extraction process (WBEP), etc. [6,7]. For example, hot alkali water washing is used primarily to separate water-wet oil sands and disadvantages include high water consumption, incomplete separation, environmental pollution from tailings, and bitumen that needs to be refined [8,9]. As the most successful and widely used technology, the WBEP brings considerable economic benefits but also a huge burden on the environment and a lot of irreparable damage, such as the consumption of energy and fresh water, the rapid expansion of tailings ponds, and the destruction of vegetation and aquatic organisms [10]. In addition, the scope of its applicability is limited by the wettability and adsorption form of the oil sands surface, and the bitumen obtained by this method contains so many solids (10% or more) that it needs to be separated twice, which endangers the future of oil sands [11,12].

Compared with the WBEP, SEP may have some disadvantages in the cost of non-aqueous reagents, but it also has considerable advantages [13]. First, this method has advantages in energy and equipment costs due to the normal operating temperature and pressure. Second, without the use of fresh water and chemical additives, efficient solvents are sufficient to obtain clean tailings and bitumen, which simplifies the tailings and bitumen purification process and eliminates the problems of wastewater treatment. Third, this method has good general applicability to oil sands due to its insensitivity to bitumen content, particulate content, and oil sands’ wettability. In recent years, the development of equipment technology and the continuous increase in petroleum prices have made it possible to achieve the commercial application of the SEP. SEP is regarded as a potentially effective alternative to the WBEP [14].

The SEP uses a similar solubility principle of organic solvents to dissolve the bitumen components on the surface of oil sands in organic solvents with strong polarity, while the clay and minerals in oil sands sink to the bottom of the solution under the action of gravity to procure the separation of oil sand asphalt and sand [15]. The strong polar solvent can not only dissolve the relatively weak polar components in the oil sands bitumen, but also diffuse into the asphaltene layer, and the asphaltene is separated from the associative shape into a loose shape. Under the action of molecular forces, the asphaltenes enter the main phase of the solvent and accelerate the separation of the oil sands [16]. The oil sands bitumen component extracted by organic solvent also has a polarity that can be used as a solvent to participate in the oil sand bitumen extraction process and promote the oil sands separation [17]. The selection of the organic solvent has a crucial influence on the separation efficiency of oil sands bitumen when using SEP; moreover, the extraction rate of oil sand bitumen is positively correlated with the content of asphaltenes in the oil sand bitumen. When the solubility parameter of the solvent is similar to that of the asphaltene, the extraction rate of oil sand bitumen is generally high [18]. The extraction efficiency depends on many factors, the most important of which are the type of solvent and the specific operating processes. The most commonly used solvents are naphtha ethers, short-chain alkanes (less than C_8_), cycloalkanes, aromatics, etc. Based on the study and analysis of raw materials properties, petroleum ether, n-hexane, xylene, n-heptane, cyclohexane, toluene, and ethyl alcohol were selected as extractants for oil sands extraction.

In addition to the selection of organic solvents, the SEP operating conditions are also an important factor affecting the separation efficiency of oil sand bitumen, including particle size, solvent—sand ratio, temperature, time, stirring speed, etc. [19]. When the extraction temperature increases, the viscosity of oil sand bitumen and the adhesion with sand particles decrease, and the mass transfer rate increases, improving the solvent extraction efficiency. For example, under stirring conditions, the extraction effect is more obvious, but the temperature cannot be too high. Otherwise, the volatilization of the solvent will be accelerated, the effective mass of the solvent will be reduced, and thus the extraction effect of the solvent will be reduced; the length of extraction time is closely related to other operating conditions. At the same time, the economy under industrial conditions should be considered when selecting the best process operating conditions [20,21].

The objective of the present work is to explore a method of SEP for the separation of oil sands. Indonesian oil sands are typical oil-wet and weathered oil sand ores (connate water less than 1 wt%) [1], and the SEP is more suitable for the separation of Indonesian oil sands [22]. The basic properties of Indonesian oil sands were examined, and the SEP was applied in bitumen extraction from oil sands. Various organic solvents were selected to investigate the extraction effects and determine suitable operating conditions. Moreover, the compositions and structures of the bitumen obtained from oil sands were analyzed so that the SEP can be better implemented for commercial applications.

## 2. Materials and Methods

### 2.1. Reagents and Materials

The high-grade Indonesian oil sands with a bitumen content of about 25% are black and brown. Since the diameter of the oil sands lump will greatly affect the extraction rate, the uneven chunks of oil sands were smashed and sieved to obtain oil sands samples with dp < 40 mesh, stirred evenly to control the variables, and sealed for storage finally [23].

Petroleum ether, xylene, n-heptane, (AR, Shanghai Lingfeng Chemical Reagent Co., Ltd., Shanghai, China); neutral alumina, cyclohexane, n-hexane (AR, Jiangsu Qiangsheng Functional Chemical Co., Ltd., Suzhou, China); tetrahydrofuran, deuterated chloroform, toluene, ethyl alcohol (AR, Sinopharm Chemical Reagent Co., Ltd., Shanghai, China).

### 2.2. Experimental Methods

The bitumen content and the water content were determined by the Dean—Stark toluene extraction method [24,25].

The 100 mL solvent was placed in a 250 mL three-necked flask with 20.0 g of oil sand samples. The process was carried out at the extraction temperature of 40 °C heated by a water bath and stirred for 30 min at 500 r/min using JJ-1B electric blender. The upper liquid was sucked into the centrifuge tube by a disposable pipette and centrifuged at 4000 r/min for 10 min; then, the supernatant in the centrifuge tube was transferred into the conical flask (*m*_0_) with a disposable pipette. Finally, the solvent in the conical flask was distilled and recovered; after that, the conical flask was dried in the vacuum and weighed (*m*). The extraction rate (*η*) was calculated as follows.

(1) $$\eta =\frac{m-m_{0}}{20.0}\times 100\%$$

### 2.3. Analyses

As the key to affecting the separation of oil sands and the features of the products, research on the chemical compositions and internal molecular structures is particularly important [26].

Bitumen is a complicated heavy oil composed of non-polar alkanes, naphthenes, and aromatic hydrocarbons with a certain polarity, and highly polar non-hydrocarbons, which makes it unsuitable for analysis from a single level only. From the perspective of polarity, liquid—solid adsorption chromatography can be used to separate bitumen into sub-fraction saturates, aromatics, resins, and asphaltenes (SARA) so that more detailed information can be obtained accurately. Firstly, the asphaltene was separated by n-heptane, and then the other three fractions were washed successively from the alumina adsorption column with petroleum ether, toluene, and toluene-ethanol (1:1) in turn (refer to SH/T0509-2010).

The separated SARA are still complex mixtures. It is generally believed that the average structural parameters, such as average molecular weight (*MW_n_*), atomic *H/C* ratio (*N_C_/N_H_*), aromaticity (*f_A_*), and Branchiness Index (*BI*) are the most appropriate methods to characterize them up until now [27]. These properties were characterized by the following methods: The *MW_n_* was measured by gel permeation chromatography (Waters Breeze GPC, Waters Corporation, Milford, MA, USA) at a ratio of samples to tetrahydrofuran in 1 g/1000 mL. The C/H/O/N/S content and *N_C_/N_H_* were analyzed by using an elemental analyzer (Vario EL III, Germany Elementar Company, Langenselbold, Germany). The AVANCE III 400M NMR (Bruker, Billerica, MA, USA) was used to measure ^1^H NMR and ^13^C NMR spectra with deuterated chloroform as solvent. About 20 mg of the sample was placed in a nuclear magnetic tube, and about 0.4 mL of deuterated chloroform reagent was added and shaken evenly. The spectral data were processed by MestRe Nova (v14.0) with a computer.

**Figure 1:**
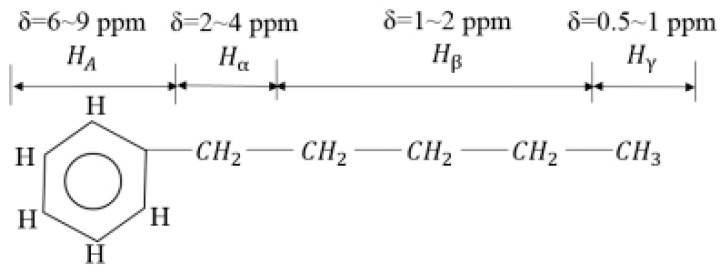
Assignments for ^1^H chemical shifts.

The *f_A_* and *BI* were calculated from the above data. The specific calculations are as follows [28]:

Assignments for ^1^H chemical shifts and formulas for *f_A_* and *BI* in ^1^H NMR spectra (Figure 1).

(2) $$f_{A}=\frac{\frac{N_{C}}{N_{H}}-\left(H_{\alpha }+H_{\beta }+H_{\gamma }\right)/2H_{T}}{N_{C}/N_{H}}$$

(3) $$BI=\frac{S_{CH_{3}}/3}{S_{\left(CH_{2}+CH\right)}/2}=\frac{H_{\gamma }/3}{\left(H_{\alpha }+H_{\beta }\right)/2}$$

where *H_A_*, *H_α_*, *H_β_*, *H_γ_* are the proportions of protons in specific molecular environments (the corresponding chemical shifts) to the total number of protons, and *H_δ_* is 1.

**Figure 2:**
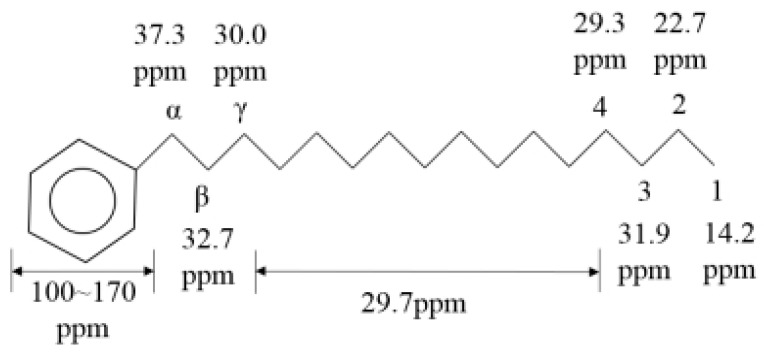
Assignments for ^13^C chemical shifts.

Assignments for ^13^C chemical shifts and formula for *f_A_* in ^13^C NMR spectra (Figure 2):

(4) $$f_{A}=\frac{A_{A}}{A_{A}+A_{S}}$$

where *A_A_* and *A_S_* are areas of aromatic carbons (δ = 100~170 ppm) and aliphatic carbons (δ = 0~70 ppm).

## 3. Results

### 3.1. Analysis of Indonesian Oil Sands

The basic properties of Indonesian oil sand samples were measured and the results are shown in Table 1. It can be seen from Table 1 that Indonesian oil sands have the characteristic of high bitumen content compared to most oil sands. Compared to Syncrude, one of the biggest oil sands producers, Indonesian oil sands with small water content, as oil-wet or weathered oil sands, have profound research value similarly since a large part of the proven oil sands resources belong to oil-wet oil sands in many countries in the world. The density of Indonesian oil sand bitumen is 1.0501 g·cm^−3^ (20 °C), and the sulfur content is 2.3 wt%, which belongs to high-sulfur heavy oil. The viscosity is 50.25 mm^2^·s^−1^ (100 °C), and the flash point and freezing point are 65 °C and 10 °C, respectively. It is not difficult to find that Indonesian bitumen has a high proportion of asphaltenes, which makes the study of oil sands separation more challenging [29,30,31,32].

### 3.2. Effect of Different Organic Solvents on the Extraction Rate of Bitumen

**Figure 3:**
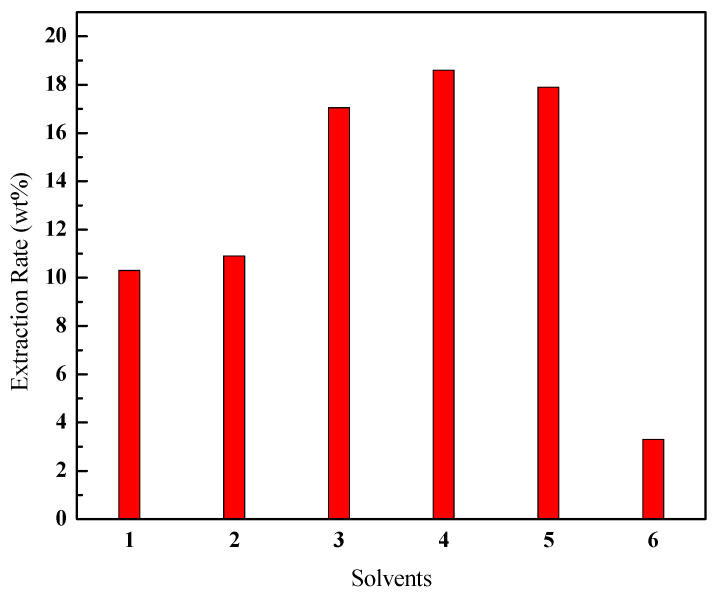
Effect of different organic solvents on the extraction rate of bitumen (1-petroleum ether; 2-n-hexane; 3-cyclohexane; 4-toluene; 5-xylene; 6-ethyl alcohol).

The effects of typical solvents, such as linear alkanes (petroleum ether, n-hexane), cycloalkanes (cyclohexane), aromatic hydrocarbons (toluene, xylene), and alcohols (ethyl alcohol), are shown in Figure 3. The extraction rates of bitumen were obtained as follows: aromatic hydrocarbons > cycloalkanes > linear alkanes > alcohols. The SEP utilizes the principle of “similar compatibility”, that is, the closer the structure and polarity of the selected solvent to the solute, the better the extraction effect. The polarities of petroleum ether and n-hexane are weak, so it was almost impossible to dissolve the highly polar asphaltene, resulting in an unsatisfactory extraction effect. The alcohols with high polarity could not dissolve the macromolecules in the oil sands, so the extraction rate was very low. Cyclohexane, toluene, and xylene have moderate polarities and are easily compatible with aromatic and naphthenic rings in resin and asphaltene, which led to high extraction rates, and toluene was optimal among these solvents; therefore, aromatic hydrocarbons and cycloalkanes were preferred in the choice of solvents, and toluene was selected as the extractant.

### 3.3. Study of Operating Conditions

#### 3.3.1. Effect of the Ratio of Toluene to Oil Sands

**Figure 4:**
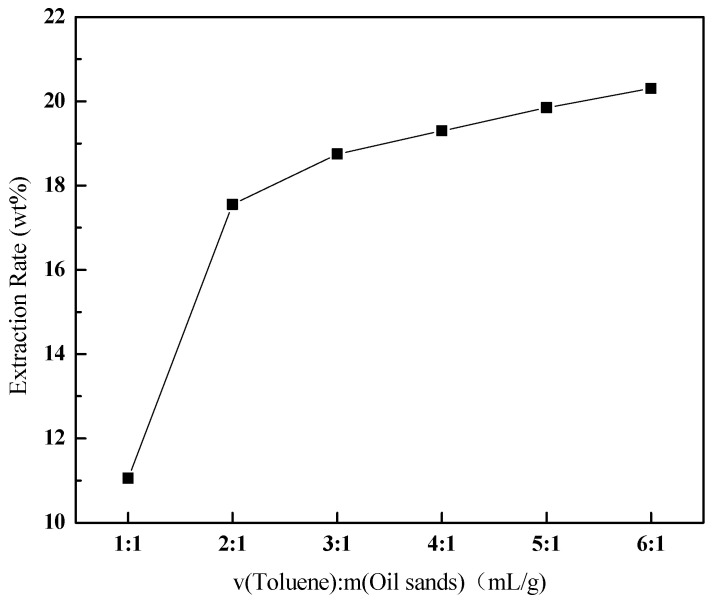
Effect of the ratio of toluene to oil sands on the extraction rate of bitumen.

The ratio of toluene to oil sands directly determines the degree of contact between toluene and oil sands, the equilibrium of bitumen resolution, and the viscosity of the solution system formed at the end, affecting the extraction rate and cost. The results (Figure 4) demonstrated that the extraction rate of bitumen increased from 11.05% to 20.3% with the increase in the ratio, under certain operating conditions (500 r/min, 40 °C, 30 min). The extraction rate rapidly increased at first, and then slowly. Because the oil sands can accommodate a fixed volume of liquid and a small ratio result in little liquid, the extraction rate is affected by both the liquid volume and the solution concentration. When the ratio is large enough, it is mainly affected by the solution concentration, and the smaller solution viscosity will also reduce the amount of clay inclusions, resulting in a rapid decrease in the rate of extraction. Considering the cost, 3:1 is suggested as a suitable toluene-to-oil sands ratio. Simultaneously, the smaller solution viscosity solved the problem of suspending solid particles in bitumen.

#### 3.3.2. Effect of the Stirring Velocity

**Figure 5:**
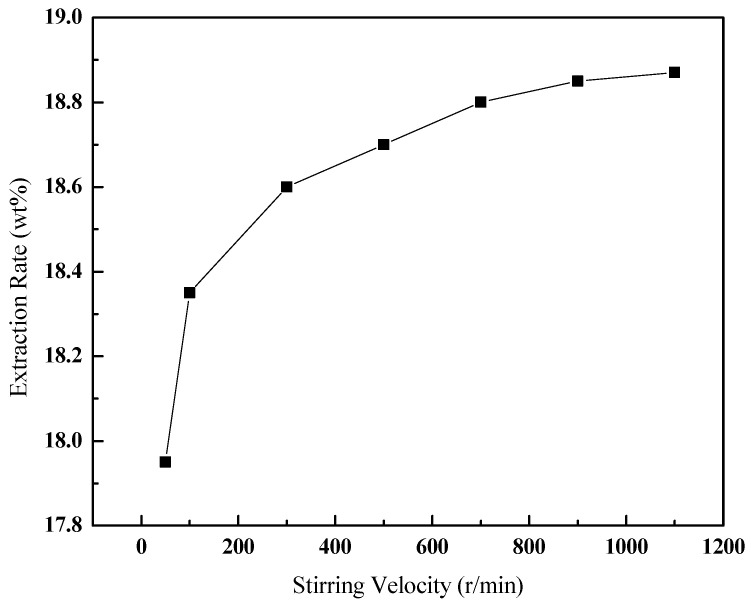
Effect of the stirring velocity on the extraction rate of bitumen.

The contact and mass transfer between toluene and oil sands can be enhanced by stirring, thereby increasing the extraction rate. The effect of the stirring velocity on the extraction rate was carefully examined under certain operating conditions (v (toluene):m (oil sands) of 3:1, 40 °C, 30 min), as shown in Figure 5. When the stirring velocity is between 50 and 300 r/min, the increase in the stirring velocity had a significant effect on the extraction rate. After 300 r/min, the extraction rate increased slowly. Considering that the stirring velocity in industrial applications cannot be as high as the velocity in the laboratory, 300 r/min is suggested as a preferable stirring velocity.

#### 3.3.3. Effect of the Extraction Temperature

**Figure 6:**
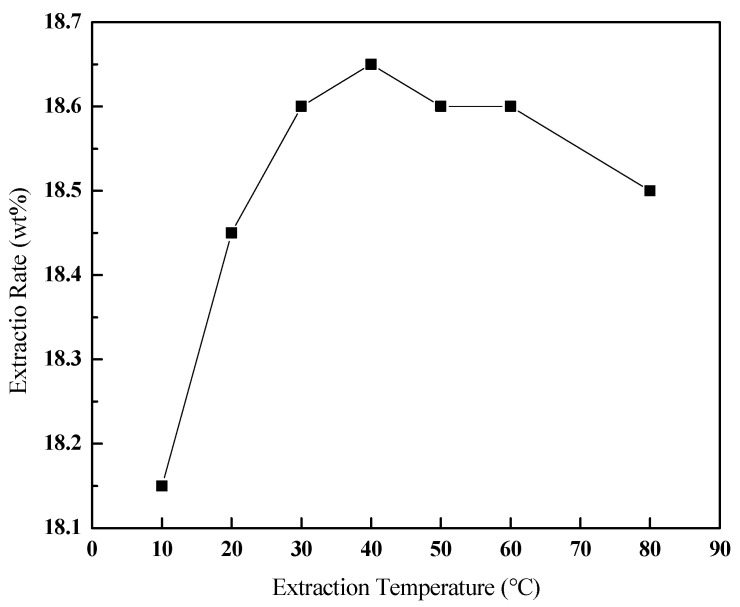
Effect of the temperature on the extraction rate of bitumen.

The temperature can affect the viscosity of bitumen, the mass transfer rate, and the solubility of bitumen in the solvent, thus affecting the extraction rate of bitumen. As shown in Figure 6, after 40 ℃, the extraction rate decreased instead of increasing greatly with the increase in temperature, under certain operating conditions (v (toluene):m (oil sands) of 3:1, 300 r/min, 30 min). The increased temperature increases mass transfer and decreases adhesion between bitumen and oil sand before 40 ℃. After that, the high temperature makes the gasification of the toluene increase, and the amount of macromolecules dissolved in toluene decreases due to the low viscosity. Hence, 40 ℃ is advised to be a suitable extraction temperature.

#### 3.3.4. Effect of the Extraction Time

**Figure 7:**
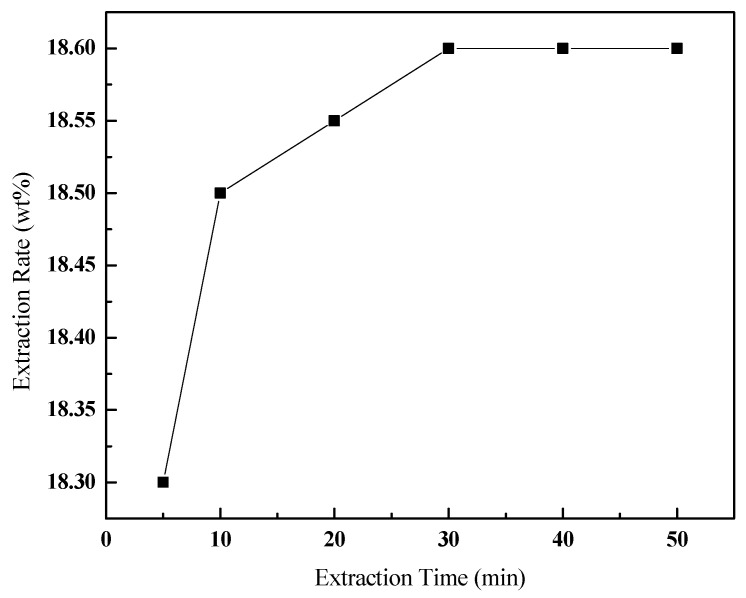
Effect of the extraction time on the extraction rate of bitumen.

Sufficient extraction time will ensure full contact and mass transfer between oil sands and toluene. The effect of the extraction time on the extraction rate is shown in Figure 7, under certain operating conditions (v (toluene):m (oil sands) of 3:1, 300 r/min, 30 ℃). As seen, the extraction rate did not increase with the extraction time increasing from 30 to 50 min, which indicates that the mass transfer process between oil sands and solvent reached a state of dynamic equilibrium after 30 min. Therefore, 30 min can be seen as a reasonable extraction time.

### 3.4. Analysis of Bitumen Obtained under Suitable Conditions

**Figure 8:**
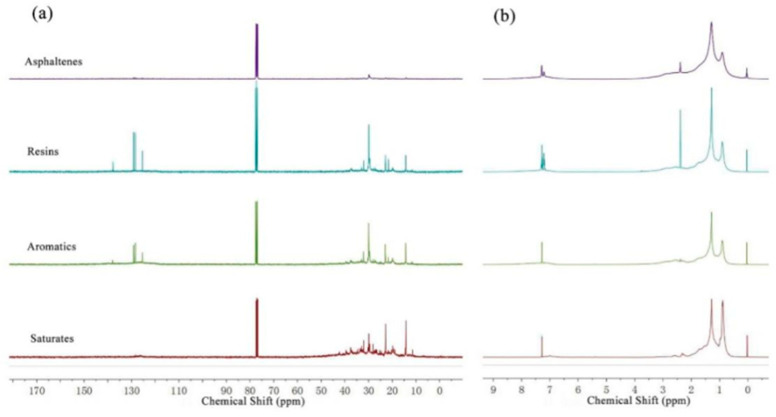
^1^H NMR and ^13^C NMR spectra of four fractions: (**a**) ^1^H NMR; (**b**) ^13^C NMR.

^1^H NMR and ^13^C NMR spectra of four fractions are shown in Figure 8. It can be visually found in Figure 8a that the *H_γ_* was the largest, and *H_A_* was the smallest in the saturates, comparatively speaking. Multiple absorption peaks appeared in the resins and asphaltenes at δ = 6~9, which proves the existence of many kinds of aromatic protons. As shown in Figure 8b, the spectrum of asphaltene was dissatisfactory because of its large molecular weight or small solubility. The chemical shifts of naphthenic carbons are generally around 20~30 ppm, and the chemical shifts of open-chain alkane carbons are less than 50.

Combined with the relationship between chemical shift and chain length, it can be inferred that there were many long chains in the saturates compared with other SARA groups. The proportions of protons and areas of carbons in specific molecular environments were obtained by MestRe Nova directly. The fundamental data are given in Table 2. The *MWn* of resins and asphaltenes increased significantly, in contrast to saturates and aromatics, with only a little difference. In terms of elemental analysis, compared with the saturates with few heteroatoms, the sulfur contents of the others were relatively high, especially asphaltenes, and the nitrogen contents of resins and asphaltenes increased rapidly. The content of oxygen increases gradually from saturates to asphaltenes.

**Figure 9:**
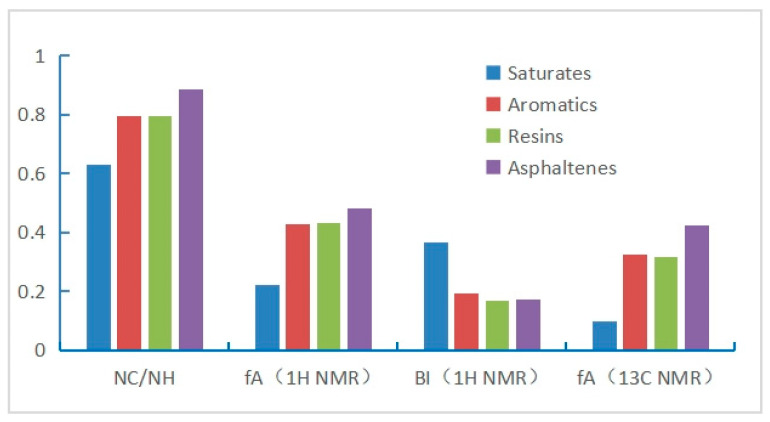
The average structural parameters of four fractions.

According to Table 2, the hydrogen—carbon atom ratio (*N_C_/N_H_*), aromaticity (*f_A_*), and branching index (*BI*) were calculated and plotted as a histogram shown in Figure 9. It is easy to compare and analyze within and between groups in the diagram. Firstly, the saturates are analyzed. It is not difficult to find that their aromaticity and hydrocarbon atom ratio are the smallest and the *BI* is the largest. It can be seen that their molecules are not only small but also there are many branches on their chains. The low aromaticity is caused by a small number of heteroatoms and cycloalkanes. The aromatics and resins of the various data are relatively close, so their physical properties are similar. Asphaltenes have the highest aromaticity and the largest hydrocarbon atom ratio, but the branching index is moderate.

The structural properties of the saturate fraction are relatively simple, and it is most easily separated from the oil sands. Aromatics and resins with similar structures can be regarded as a whole component in the separation process of industrial oil sands. To focus on the analysis of the most complex structure of the asphaltenes, the industrial production process can also try to separate the other three components, and then separate them from the asphaltenes, to develop new oil sand utilization.

For oil-wet oil sands, oil sand bitumen penetrates the gaps in solid sands and is wrapped on the outer surface of solid sands. When the oil sand particles are small, the specific surface area of the oil sand particles increases, which means that there is a larger contact area with the organic solvent; however, the oil sand particle size cannot be too small. If the small oil sand particles are blindly pursued, the sand rock mass will be broken, which will be unfavorable to the separation. In the case of wetting, these ultrafine particles are easily emulsified with oil sand bitumen, water, etc., forming emulsions, which will have an adverse effect on oil sand separation.

From the perspective of sub-fractions, SARA fractions were not gradually or homogeneously distributed in the bitumen layer of oil sands. Saturates and aromatics tend to be distributed in the outer layer of the bitumen of the sand surface. The relative content of weak polar components (saturates and aromatics) in the outer surface of oil sand bitumen is higher, while the relative content of strong polar components (resins and asphaltenes) is lower. Oppositely, resins and asphaltenes tend to be distributed in the inner layer of the bitumen. In the inner layer of oil sand bitumen, the relative content of strong polar components is higher, and the relative content of weak polar components is lower. The extraction solvent molecules diffuse to the outer layer of the surface of the oil sand bitumen, dissolving the less polar components in the oil sand bitumen, reducing the viscosity of the bitumen, and thereby reducing the adhesion between the oil sand bitumen and the sand particles. On this basis, the extraction solvent molecules can further diffuse into the strong polar resins and asphaltene layer in the inner layer, and resin and asphaltene are separated from the associative shape into a loose shape [33]. Under the action of molecular force, they enter the main phase of the solvent, thus accelerating the separation of bitumen and oil sand solid sand.

## 4. Discussion

Under the above optimum conditions, the extraction rate of oil sand oil by toluene can reach 18.55%. It was found that the tailings oil content can still reach about 7.5% after toluene extraction, which is not enough to meet the national requirements for tailings discharge standards. According to the formula of material conservation, the oil content of Indonesian oil sands is 24.93%. Only when the extraction rate reaches 24.7%, the oil content of tailings is less than 0.3%, which meets the discharge standard of tailings. The analysis of the influence of the above process conditions on the extraction rate shows that increasing the solvent—sand ratio is an effective way to improve the extraction rate. To achieve the extraction rate of 24.7%, an extremely large solvent—sand ratio is needed, which will greatly increase the economic cost. Under the above suitable process conditions, the experiment of multiple extractions found that the extraction rate of oil sand oil was 23.03% by twice extraction. After three times of extraction, the extraction rate of oil sand oil was 24.72%, and the tailings also reached the emission standard, but the ratio of solvent to sand was too large, and the extraction process needed to be improved again.

Indonesian oil sands contain a large number of asphaltenes and resins with high polarity and complex structures. The complex chemical formulation of bitumen makes the characterization processes difficult. The physical and chemical properties of bitumen are highly affected by the proportion of SARA. The strong polar fractions, such as asphaltenes and resins, with a stronger adhesion force with sand grains, tended to be distributed in the inner bituminous layer due to their higher polarity, while the weak polar fractions such as saturates predominated the outer layer. The molecular weight of the saturates is small and the branching index is extremely high, indicating that they are mainly small molecular alkanes and cycloalkanes containing branched chains, and can be easily separated. The molecular weight and nitrogen content between aromatics and resins are much different, and other parameters are close, indicating that resins are larger than aromatics and have many amino groups, while their physical properties are very similar. The colloidal structure, strength, and stiffness of bitumen are affected by the number of asphaltenes. The molecular weight of asphaltenes of oil sand bitumen is much larger than that of other components, and the carbon—hydrogen atomic ratio, aromatic carbon ratio, and heteroatom of asphaltenes are also much higher than those of other components. Therefore, the performance of asphaltene should be studied emphatically in the process of oil sand oil separation and application.

At present, oil sands are developing rapidly in the direction of scale and commercialization. The SEP method has the advantages of a high bitumen extraction rate, a simple process, and no water participation; the disadvantage is that the one-time investment is large, which easily can cause solvent waste and secondary environmental pollution. At the moment, the problems to be solved in the development and utilization of oil sand resources are to reduce the mining cost, improve the bitumen recovery efficiency, and select economical and effective solvents to extract bitumen from oil sands. The cost of solvent will play a vital role in the oil sands industry on a commercial scale. Therefore, emphasis on researching the suitable solvent can lead to the process being environment-friendly and cost-effective.

## 5. Conclusions

In this study, a method of solvent extraction for the separation of Indonesian oil sands was shown to be an efficient technique. The separation performance was affected by different organic solvents and operating conditions. It was shown that the closer the structure and polarity of the selected solvent is to the solute, the better the extraction effect. The operating conditions were also important factors affecting the separation effect of oil sand bitumen, including the ratio of toluene to oil sands, stirring velocity, extraction temperature, and extraction time. It was also found that Indonesian oil sands were oil-wet oil sands with a bitumen content of 24.93%. The extraction rate of bitumen could be 18.55% using toluene as the extraction solvent under the operating conditions of V (solvent):m (oil sands) 3:1, temperature 40 °C, stirring velocity 300 r/min, and time 30 min. The advantage of this method is that bitumen can be separated and analyzed efficiently from the complex oil sands, and the method may be applied to the separation of other oil-wet oil sands. The compositions and structures of the bitumen can provide guidance for the separation and comprehensive utilization of industrial oil sands.

## Figures and Tables

**Table 1:** Basic properties of Indonesian oil sands and bitumen.

Property		Indonesian Samples	Syncrude Canada Ltd.	Neimenggu China
Oil Sands	Bitumen	24.93	8~15	12~20
	Solids	74.30	83~88	80~87
	Water	0.77	2.5~9	0.5~1
Bitumen	Saturates	23.56	16~21	34.1
	Aromatics	23.08	26~36	13.2
	Resins	21.61	24~41	37.7
	Asphaltenes	31.75	15~24	12.8

**Table 2:** The fundamental data of four fractions.

Property		Bitumen	Saturates	Aromatics	Resins	Asphaltenes
*MWn*		802	443	573	981	1984
Elemental Analysis	C/%	80.86	86.16	81.56	80.19	76.62
	H/%	8.84	11.427	8.594	8.438	7.234
	O/%	3.071	1.983	3.014	3.143	4.328
	N/%	0.43	0.07	0.17	0.70	0.77
	S/%	6.275	0.351	6.043	6.751	9.762
^1^H NMR	*H_A_*	0.091	0.018	0.091	0.094	0.083
	*H_α_*	0.156	0.048	0.195	0.177	0.160
	*H_β_*	0.533	0.586	0.509	0.546	0.568
	*H_γ_*	0.220	0.348	0.205	0.183	0.189
^13^C NMR	*A_A_*/%	31.746	9.891	32.680	31.546	42.553
	*A_S_*/%	68.254	90.109	67.320	68.454	57.447

## Data Availability

Not applicable.
